# Crystallization Study and Comparative *in Vitro*–*in Vivo* Hydrolysis of PLA Reinforcement Ligament

**DOI:** 10.3390/ijms12106597

**Published:** 2011-10-10

**Authors:** Theodore Beslikas, Ioannis Gigis, Vasilios Goulios, John Christoforides, George Z. Papageorgiou, Dimitrios N. Bikiaris

**Affiliations:** 1 2nd Orthopaedic Department, Aristotle University of Thessaloniki, Thessaloniki, Macedonia 54124, Greece; E-Mails: beslikastheo@in.gr (T.B.); jgigis@otenet.gr (I.G.); bill.goulios@gmail.gr (V.G.); hristofo@med.auth.gr (J.C.); 2 Laboratory of Polymer Chemistry and Technology, Department of Chemistry, Aristotle University of Thessaloniki, Thessaloniki, Macedonia 54124, Greece; E-Mail: gzpap@chem.auth.gr

**Keywords:** PLA, reinforcement ligaments, orthopaedics, crystallization, *in vitro*–*in vivo* hydrolysis

## Abstract

In the present work, the crystallization behavior and *in vitro*–*in vivo* hydrolysis rates of PLA absorbable reinforcement ligaments used in orthopaedics for the repair and reinforcement of articulation instabilities were studied. Tensile strength tests showed that this reinforcement ligament has similar mechanical properties to Fascia Latta, which is an allograft sourced from the ilio-tibial band of the human body. The PLA reinforcement ligament is a semicrystalline material with a glass transition temperature around 61 °C and a melting point of ~178 °C. Dynamic crystallization revealed that, although the crystallization rates of the material are slow, they are faster than the often-reported PLA crystallization rates. Mass loss and molecular weight reduction measurements showed that *in vitro* hydrolysis at 50 °C initially takes place at a slow rate, which gets progressively higher after 30–40 days. As found from SEM micrographs, deterioration of the PLA fibers begins during this time. Furthermore, as found from *in vivo* hydrolysis in the human body, the PLA reinforcement ligament is fully biocompatible and after 6 months of implantation is completely covered with flesh. However, the observed hydrolysis rate from *in vivo* studies was slow due to high molecular weight and degree of crystallinity.

## 1. Introduction

Biodegradable polymers have attracted a lot of attention over the last two decades due to their importance from a fundamental as well as a commercial point of view. The term biodegradable is used to characterize materials that are hydrolysable at temperatures up to 50 °C (e.g., in composting) over a period of several months to one year. As a result of hydrolysis, biodegradable polymers break down in physiological environments by macromolecular chain scission into smaller fragments, and ultimately into simple stable end-products [1]. Among the numerous polyesters studied so far, poly(lactic acid), (PLA) has proven to be the most attractive and useful biodegradable polymer, which has been primarily used in biomedical applications. This is because PLA can be derived from renewable resources such as corn, potato, cane molasses and beet sugar and is biocompatible and bioabsorbable with favorable mechanical properties [2]. PLA belongs to the poly (a-hydroxy acids) group and due to asymmetric molecular structure of lactic acid, PLA exists as L-PLA (mostly crystalline) or DL-PLA (mostly amorphous). Commercially available high molecular weight PLA resins are produced via the lactide ring-opening polymerization route [3]. The mechanical properties of high molecular weight PLA are comparable to other commodity thermoplastics like polystyrene and PET, and therefore it might replace these polymers for numerous applications [4,5]. Although its high cost has limited its uses until recently, the latest technological advances have given rise to a PLA that is commercially viable and can compete with petrochemical plastics [4].

The preparation of PLA fiber began in the latter part of the 1990s. Apart from being fully biodegradable and biocompatible, PLA fibers also have other advantages, such as lower consumption of energy and resources compared with petroleum-based synthetic fibers, as well as good physical and mechanical properties. However, PLA fibers have poor hydrophilicity due to a large number of ester bonds in the molecule which can decrease its hydrolytic degradation ratio. PLA is used extensively in surgery as suture materials and bone fixation devices; a large number of investigations have been carried out on PLA and its copolymers in biomedical applications for resorbable medical implants [6–8]. Biodegradable polymers like PLA have specific advantages over metallic or ceramic materials in medical applications [9,10]. PLA can be used in the shape of a rod, plate, screw, fiber, sheet, sponge, or beads for bone and tissue engineering, or as microspheres for drug delivery systems [3,11]. PLA has been used for applications like biodegradable/bioabsorbable fibrous articles for medical applications [12,13] and orthopaedics screw [14]. The other biomedical applications of PLA include the development of scaffolds [15], biocomposite material [16], sutures [17], prosthetics, *etc.* Moreover, low molecular weight PLA is used in tissue engineering [18,19].

PLA degrades by simple hydrolysis of the ester bond and it does not require the presence of enzymes to catalyze hydrolysis [4]. When PLA hydrolyzes, it forms lactic acid, a normal product of muscle contraction. Lactic acid also enters the tricarboxylic acid cycle and is excreted as water and carbon dioxide. Thus, the degradation products of PLA are non-toxic to the living organisms since lactic acid itself occurs in the metabolism. Degradation of PLA is dependent on time, temperature, low-molecular weight impurities, and catalyst concentration [5]. Based on available data to date, the duration of degradation can range from 12 months to over 2 years [5].

Degradation of aliphatic polyesters has been extensively studied [20–23], and mathematical models have been developed to predict the hydrolytic degradation process [24,25]. However, the *in vivo* behavior of the PLA reinforcement ligament, especially in the human body, has never been described before and published papers report only on neat PLA or other materials [4,26,27]. In this work, commercial PLA absorbable reinforcement ligament for the repair and reinforcement of articulation instabilities from Cousin Biotech under the trade name Resorbaid^®^ was studied. The Resorbaid flat braids are used for intra or extra-articular implantation and reinforcement of articular plastia of the knee, the shoulder, or the ankle. The study included crystallization kinetics and *in vitro–in vivo* hydrolysis experiments. To the best of our knowledge, this is the first time that *in vivo* hydrolysis results from tests in humans are reported.

## 2. Materials and Methods

### 2.1. Materials

Commercial reinforcement ligament consisted from PLA supplied under the trade name Resorbaid^®^ from Cousin Biotech (France). Fascia Latta human allograft was supplied from Tubogen Medical GmbH, a RTI Biologics^TM^ company. All other materials and solvents used were of analytical grade and have been purchased from Aldrich.

### 2.2. Measurements

#### 2.2.1. Intrinsic Viscosity

Intrinsic viscosity [*η*] measurements were performed by using an Ubbelohde viscometer at 25 °C. All polyesters were dissolved in chloroform at room temperature in order to prepare solutions with concentrations up to 1 wt% and filtered through a disposable membrane filter 0.2 μm (Teflon). Intrinsic viscosity was calculated after the Solomon-Ciuta equation [28]:

(1) $$[\eta ]={\left\{2[t/t_{o}-\text{ln}(t/t_{o})-1]\right\}}^{1/2}\;c^{-1}$$

where *c* is the concentration of the solution; *t*, is the flow time of solution and *t_o_* the flow time of pure solvent.

#### 2.2.2. Gel Permeation Chromatography (GPC)

Molecular weight determinations were performed by gel permeation chromatography (GPC) method using a Waters 150 C GPC equipped with differential refractometer as detector and three ultrastyragel (103, 104, 105 Å) columns in series. Tetrahydrofuran (THF) was used as the eluent (1 mL/min) and the measurements were performed at 35 °C. Calibration was performed using polystyrene standards with a narrow molecular weight distribution.

#### 2.2.3. Scanning Electron Microscopy (SEM)

The morphology of PLA reinforcement ligament before and after hydrolysis was examined in a scanning electron microscopy (SEM) type Jeol (JMS–840) equipped with an energy-dispersive X-ray (EDX) Oxford ISIS 300 micro-analytical system.

#### 2.2.4. Mechanical Properties

Measurements of tensile mechanical properties of PLA reinforcement ligament and Fascia Latta samples were performed on an Instron 3344 dynamometer, in accordance with ASTM D638, using a crosshead speed of 5 mm/min. The values of Young’s modulus, yield stress, elongation at break and tensile strength at the break point were determined. At least five specimens were tested for each sample and the average values, together with the standard deviations, are reported.

#### 2.2.5. Differential Scanning Calorimetry (DSC)

Thermal behavior of the PLA ligament was studied using a Perkin-Elmer Pyris Diamond DSC differential scanning calorimeter. The instrument was calibrated with high purity indium and zinc standards. Samples of about 5 mg were used.

It is important for the crystallization experiments to minimize the thermal lag, so low mass samples should be used in crystallization tests. For isothermal crystallization tests the samples were first melted to 220 °C for 3 min to erase any previous thermal history and then cooled to the crystallization temperature (*T*_c_) at a rate 200 °C/min. The samples were held at *T*_c_ till the end of crystallization. For non-isothermal crystallizations from the melt, the samples were first melted to 220 °C for 3 min and then cooled to 25 °C at a various cooling rates, namely 2.5, 5, 7.5, 10, 15 and 20 °C/min. For non-isothermal crystallizations from the glass to record cold-crystallization, the samples were first melted to 220 °C for 3 min and then cooled to 25 °C at a rate 200 °C/min. Subsequently, heating scans at rates 2.5, 5, 7.5, 10, 15 and 20 °C/min were performed in the range from 25 to 220 °C. It should be noted that the PE Pyris Diamond DSC used for raw data collection in this work, is an instrument with low mass furnace and high sensitivity, appropriate for quite accurate measurements. A Perkin-Elmer Intracooler II was used to allow the DSC to achieve high and constant cooling rates.

#### 2.2.6. Polarizing Light Microscopy (PLM)

A polarizing light microscope (Nikon, Optiphot-2) equipped with a Linkam THMS 600 heating stage, a Linkam TP 91 control unit and also a Jenoptic ProgRes C10plus camera with the Capture Pro 2.1 software was used for PLM observations.

#### 2.2.7. *In Vitro* Hydrolysis

PLA reinforcement ligament in the form of thin films was placed in Petrie dishes containing phosphate buffer solution (pH 7.2). The dishes were then incubated at 50 ± 1 °C in an oven for several days. After a specific period of incubation, the samples were removed from the Petrie dishes, washed with distilled water and weighted until constant weight. The degree of enzymatic hydrolysis was estimated from the mass loss, molecular weight measurements, SEM analysis and DSC scans. In this case (DSC) the samples were heated in an inert atmosphere with a heating rate 20 °C till 190 °C, quenched rapidly to 20 °C in order to become amorphous and rescanned again till 190 °C.

#### 2.2.8. *In Vivo* Hydrolysis

PLA reinforcement ligament were use for ligament reconstruction in 12 patients with ankle and tibia tubercle fractures that were treated with internal osteosyntheses. The ligaments that were reconstructed were the deltoid ligament of the ankle and the patellar tendon ligament in the knee. Twelve months postoperatively the internal fixation was removed from a patient in a second operation and the reinforcement ligament was studied.

## 3. Results and Discussion

### 3.1. Characterization of PLA Reinforcement Ligament

**Figure 1:**
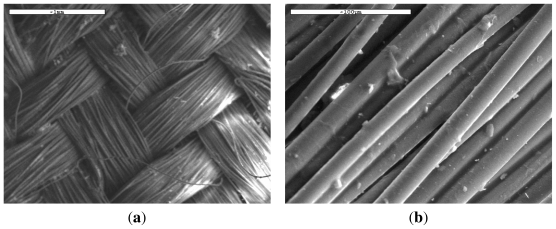
SEM micrographs with (**a**) low and (**b**) high magnification showing texture of Resorbaid poly(lactic acid) (PLA) reinforcement ligament.

Resorbaid^®^ reinforcement ligaments are made of poly(l-lactic acid) which is a bio-compatible material and fully bio-absorbable in the long term. The polyester has an intrinsic viscosity 1.41 dL/g. This corresponds to a high average number molecular weight (Mn) 120,000 g/mol which is necessary in order to prepare reinforcement ligaments with high mechanical properties. The ring opening polymerization of PLA is an important method to obtain such high molecular weight products, in which using high purity lactide is the most important factor in the whole process. The reinforcement ligament is designed for the repair and the reinforcement of articulation instabilities and can be fully desorbed in the human body within approximately 3–5 years. The ligament structure, which can be seen in Figure 1(a), entails a quick in-growth of fibrosis and an excellent tissue re-colonization. Due to its design, consisting of fibers with an average diameter 15 μm (Figure 1(b)), and its faultless mechanical properties, it enables improvement in the primary mechanical resistance of the articular ligament during the first 6 months.

**Figure 2:**
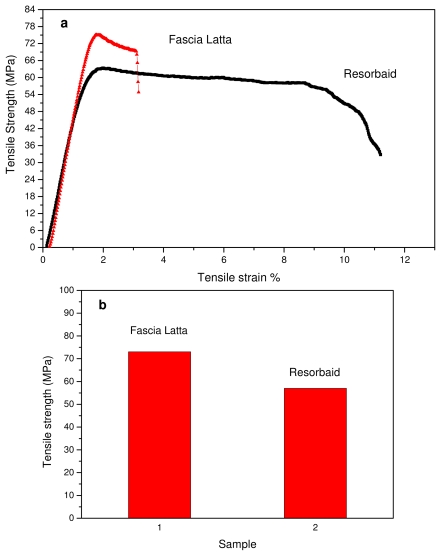
(**a**) Stress-strain curves of Fascia Latta and Resorbaid and (**b**) Tensile strength of these materials.

Tensile properties of the Resorbaid compared with Fascia Latta specimens were measured using an Instron tensile testing machine. Fascia Latta is a flexible natural collagen tissue scaffold that allows for new host tissue organization along its native fibers. From stress-strain curves (Figure 2(a)) it is clear that Fascia Latta breaks almost immediately after yielding, while Resorbaid has slightly higher elongation at break (about 10%). However, both materials can be characterized as brittle and strong since they have high tensile strengths. In fact, Resorbaid shows a tensile strength of 57 MPa compared to 73 MPa of Fascia Latta. This is very important since, in orthopaedic applications such as reinforcement ligaments, the material may be subjected to significant loads. The polymer’s molecular weight affects its mechanical properties and degradation behavior and is therefore a critical property to evaluate. It was found that, in order to perform as a load-bearing orthopaedic implant, a L-PLA polymer with a molecular weight of at least 100 kDa should be used [29,30]. For this reason, the PLA used for the preparation of Resorbaid has a number average molecular weight about 120,000 g/mol. However, this high molecular weight can affect the hydrolysis rate of the reinforcement ligament.

### 3.2. Crystallization Studies

In addition to molecular weight, the degree of crystallinity and crystallization rates can have an effect on the hydrolysis rate of PLA. Thus, crystallization studies on PLA ligament are very important since, as previously reported, further crystallization can take place during *in vivo* hydrolysis and this can affect the degradation rates of PLA [4,31]. During hydrolysis, PLA oligomers can be formed due to the ester bond cleavage as well as acidic end groups. This results in the formation of a skin composed of a polymer which degrades less rapidly than the polymer located away from the surface. Thus, it can be said that the surface of PLA fibers is less susceptible to hydrolysis rate than the interior part. The whole mechanism can result in hollow residual structures when the matrix remains amorphous for the whole degradation process. It is critical to note that if the matrix is initially crystalline or crystallizes during degradation, the inner part of large size devices degrades faster than the surface but does not lead to hollow residual structures. However, it leads to tiny crystalline residues which can be inflammatory when they remain at the surgery site, even if they are less inflammatory than the residues issued from a quenched device [4].

**Figure 3:**
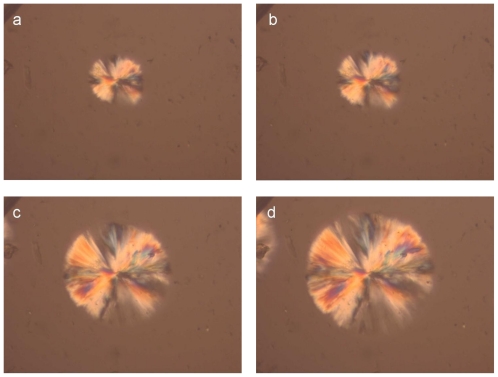
Images showing the spherulite growth during crystallization of PLA of reinforcement ligament samples at different times at 155 °C.

For a direct observation of the crystallization of the PLA reinforcement ligament and for a better understanding of related phenomena relating to aspects of nucleation and growth, polarized light microscopy was employed. Figure 3 shows photographs taken during isothermal crystallization of PLA samples cut from the reinforcement ligaments (Figure 3) at 155 °C after cooling from melt. The spherulites formed after 2 min at 155 °C and their size increased progressively with increasing isothermal crystallization time.

**Figure 4:**
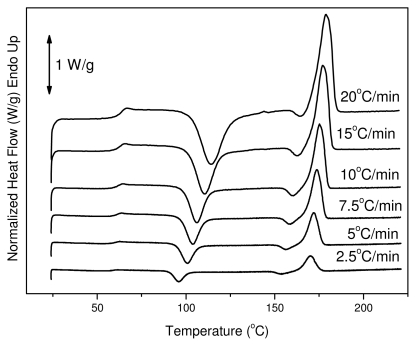
Differential scanning calorimetry (DSC) traces on heating for melt-quenched reinforcement ligament.

The reinforcement ligament consists of semicrystalline PLA, thus dynamic crystallization has also been studied after quenching from the melt. Cold crystallization of the polymers of this work was studied with DSC. Thus, heating traces of amorphous samples were recorded at various heating rates from 2.5 to 20 °C/min. In these traces it is evident that PLA has a glass transition temperature around 60 °C, which shifted upwards to higher temperatures with increasing heating rate. This is a very high value since glass transition below body temperature ensures that the material is flexible and adaptable in response to mechanical loading. The same trend can be observed for the cold-crystallization peak (*T*_cc_). At a heating rate of 2.5 °C/min the T_cc_ value is 96 °C and shifted to 115 °C at heating rate of 20 °C/min. Also, this was broadened with increasing heating rate (see Figure 4). After cold crystallization the material melts at about 170 °C.

To quantitatively describe the evolution of the relative degree of crystallinity *X* during nonisothermal crystallization, a number of models have been proposed in the literature [32]. The most common approach is that based on a modified Avrami equation [32,33]. Thus, the Avrami model can be modified to describe the crystallization kinetics under non-isothermal conditions:

(2) $$X(t)=1-\text{exp}(-Z_{t}t^{n})$$

where *Z_t_* and *n* denote the growth rate constant and the Avrami exponent, respectively.

Taking the logarithms Equation 2 can be written as:

(3) $$\text{log}\{-\text{ln}[1-X(\text{t})]\}=\text{log}Z_{t}+n\text{log}t$$

According to the Ozawa theory the non-isothermal crystallization process is the result of an infinite number of small isothermal crystallization steps and the degree of conversion at temperature *T*, *X*(*T*), can be calculated as [34]:

(4) $$\text{ln}[1-X(T)]=-\frac{K*(T)}{a^{m}}$$

where *m* is the Ozawa exponent that depends on the dimension of crystal growth and K^*^ is the cooling or heating crystallization function. K* is related to the overall crystallization rate and indicates how fast crystallization occurs. *a* is the heating or cooling rate. Taking the double-logarithmic form of Equation 4, it follows:

(5) log{-ln[1-X(T)]}=logK*(T)-mloga

Mo and co-workers [35] proposed a different kinetic model by combining the Ozawa and Avrami equations. As the degree of crystallinity was related to the cooling rate α and the crystallization time *t* or temperature *T* the relation between α and *t* could be defined for a given degree of crystallinity. Consequently, combining Equations 3 and 5 derived a new kinetic model for non-isothermal crystallization:

(6) $$\text{ln}\;Z_{t}+n\text{ln}\;t=\text{ln}K*(T)-m\text{ln}\;a$$

**Figure 5:**
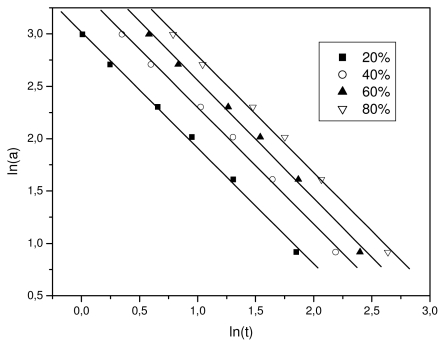
Plots constructed after Mo’s analysis for non-isothermal crystallization of Resorbaid material for various relative degree of crystallinity values.

By rearrangement at a given degree of crystallinity and solving for the cooling or heating rate *a*, Equation 6 becomes:

(7) ln a=ln F(T)-bln t

where *F*(*T*) = [K^*^(*T*)*/Z_t_* ]1*/m*, refers to the value of cooling rate chosen at unit crystallization time, when the system has a certain degree of crystallinity, *b* is the ratio of the Avrami exponent to Ozawa exponents, *i.e.*, *b = n*/*m*. According to Equation 7 at a given degree of crystallinity the plot of ln α against ln *t* will give a straight line with an intercept of ln *F*(*T*) and a slope of −*b*. As it is shown in Figure 5, plotting ln *a* against ln *t*, at a given degree of crystallinity, a linear relationship was observed (correlation coefficient *R* > 0.997). The values of *F*(*T*) and the slope *b* are listed in Table 1. The *F*(*T*) values increased with the relative degree of crystallinity. However, *b* was practically constant as it ranged from 1.47 to 1.51. In addition, these values mean that the Avrami exponent *n* is always slightly greater than the Ozawa exponent, *m*, as has also been reported in literature [32]. Non-isothermal crystallization is difficult to describe with a single equation since there are a lot of parameters that have to be taken into account simultaneously. The importance of this method is that it correlates the cooling rate to temperature, time, and morphology.

The Avrami model is suitable for describing the early stages of crystallization. Complications arise from the effects of growth site impingement and secondary crystallization process, which were disregarded for the sake of simplicity in the original derivation of the model. Tobin proposed a theory for crystallization with growth site impingement [36–38]. According to this approach, the relative crystallinity function of time X(t) can be expressed in the following form:

(8) $$X(t)=\frac{{\left(K_{T}t\right)}^{n_{T}}}{1+{\left(K_{T}t\right)}^{n_{T}}}$$

where *K_T_* and *n_T_* are the Tobin crystallization rate constant and the Tobin exponent, respectively and t is the time of crystallization. The exponent *n_T_* need not be an integer and is governed by different types of nucleation and growth mechanisms. Equation 8 can be rewritten in its logarithmic form as follows:

(9) $$\text{log}[X(t)/(1-X(t)]=\text{log}\;K_{T}+n_{T}\;\text{log}\;t$$

**Figure 6:**
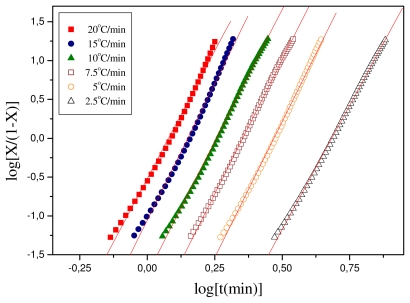
Plots constructed after Tobin’s analysis for non-isothermal crystallization of Resorbaid material.

The parameters *n_T_* and *K_T_* can be obtained from the slope and intercept of the plots of log [*X(t)/(1* − *X(t))*] against *log* t. The respective plots for the polymeric materials of this study are shown in Figure 6 and the values of the calculated parameters are shown in Table 2. For Resorbaid, the Tobin plots seem to be linear for almost the full range of crystallization. Only for fast heating rates, *i.e.*, 15 or 20 °C/min there appears some limited curvature.

In general, it can be concluded that the Resorbaid material shows slow crystallization. However, the Resorbaid material crystallization rates were found to be faster than those in most cases of PLA resins [31].

### 3.3. In Vitro Hydrolysis

**Figure 7:**
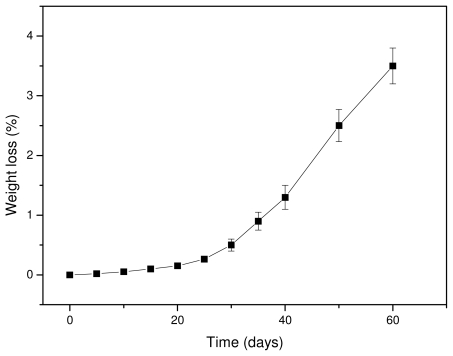
Weight loss of the PLA ligament during hydrolysis at 50 °C.

It is well known that hydrolytic scission of the macromolecular chains of aliphatic polyesters starts upon contact with water, which can hydrolyze the ester bonds. Since the hydrolysis rate of PLA is very slow, tests were performed at a high temperature (50 °C) to accelerate the phenomenon. Water can hydrolyze the macromolecular chains randomly, reducing the molecular weight and producing soluble oligomers. Thus, the hydrolysis rate can be deduced from weight loss measurements. As can be seen from Figure 7, the weight loss of Resorbaid ligament is very small at initial stages and increases dramatically after 30–40 days of hydrolysis. It seems that, at the first days of hydrolysis, only small fragments are removed, probably because the hydrolysis rate is very small or takes place at the ends of macromolecular chains and thus fragments with low molecular weights are formed. However, it was reported that the low weight loss at the initial stages of hydrolysis could be attributed to the hydrolysis of polyesters that take place randomly along the macromolecular chains, reducing only the molecular weight of the polyesters [39]. If this happens, a high molecular weight reduction should be recorded, followed by a small weight loss since the formed oligomers at initial degradation stages are too large, hence it has difficulty diffusing through the bulk material. Only after an extended period of time are they sufficiently reduced in size by hydrolysis to diffuse out as oligomers and result in a significant mass reduction [40]. Furthermore, the formation of water-soluble lactic acid oligomers is hydrolysis time-dependent. Höglund and co-workers found that pure PLA can form water soluble oligomers after 28 days of hydrolysis at 60 °C and after 133 days of hydrolysis at 37 °C [41]. The mass loss was considerably slower, and over 90% of the original mass remained in all materials after 28 days of degradation. This is due to the hydrophobic nature of PLA. The degradation products formed during hydrolysis are not water-soluble until they have a molar mass of >1,000 g/mol and therefore remain in the polymer bulk. It seems that the same also happens in our study. Thus, the mechanism of hydrolysis could be revealed in comparison with the changes in molecular weight of the PLA ligament during hydrolysis.

**Figure 8:**
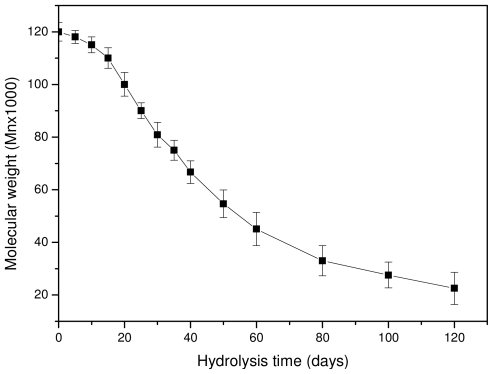
Molecular weight variation of a PLA ligament during hydrolysis at 50 °C.

As can be seen in Figure 8, the molecular weight is slightly reduced at the initial stages of hydrolysis and the rate becomes higher after 15–20 days of hydrolysis. However, after 80–100 days the rate seems to become progressively slower. The whole reduction seems to be S-type, which agrees with previously reported results [23,39]. The scission of ester bonds at initial hydrolysis times is slow, becoming progressively more rapid [42]. This can also explain the low weight loss that is recorded at the initial 15–20 days of hydrolysis. The high molecular weight of used PLA for the preparation of reinforcement ligament or its surface hydrophobicity could be responsible for such behavior [43]. Fukuzaki and coworkers found that the molecular weight reduction of high molecular weight L-lactide/glycolide copolymers becomes higher only after a certain time of hydrolysis [39]. Thus, at this time, low molecular weight oligomers are formed and this explains the high weight loss that is observed after 30–40 days of hydrolysis. These results are in disagreement with a recent study by Dånmark *et al.* [44], where it was found that, during hydrolysis, the mass loss is very small but at the same time the molecular weight reduction is very high. As can be seen in the case of this work, the weight loss reduction at the initial stages is small since less than 0.2 wt % of the initial material is lost, followed by a small reduction in molecular weight. Soluble oligomers are formed after that and the rate is higher after 35–40 days of hydrolysis. However, as can be seen in Figure 8, the high reduction in molecular weight appears at lower hydrolysis times (20 days). This is an indication that the macromolecular chains are first randomly broken during hydrolysis and, when this happens to a large extent, soluble oligomers are formed, resulting in a reduction to mass loss.

**Figure 9:**
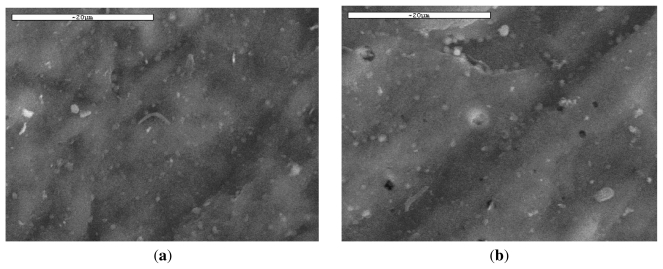

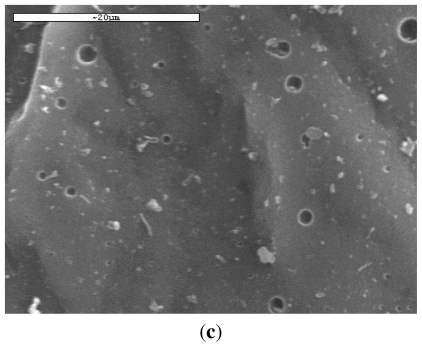
SEM micrographs of PLA ligament during hydrolysis at 50 °C for (**a**) 0 days; (**b**) 30 days; and (**c**) 60 days.

SEM was also used to study the microstructure of PLA and its variation during hydrolysis. It can been seen from Figure 9 that significant etching character appeared on the surfaces of PLA films, while the surface of blank specimen was very smooth. Although the etching effect was not uniform, it primarily demonstrated that PLA ligaments can be hydrolyzed with time. However, as has already been reported, hydrolysis of PLA ester bonds starts homogeneously until soluble oligomers are formed, which can be removed from the matrix. At this time, those soluble oligomers which are close to the surface can leach out before total degradation, whereas those located well inside the matrix remain entrapped. As can be seen, small holes were created in the PLA ligament surface after 30 days of hydrolysis. These initially have small diameter, less than 1–2 μm, which increases progressively. Thus, after 60 days of hydrolysis, the size of the formed holes is in the range of 2–5 μm. This is in agreement with the recorded weight loss and molecular weight reduction during this time.

**Figure 10:**
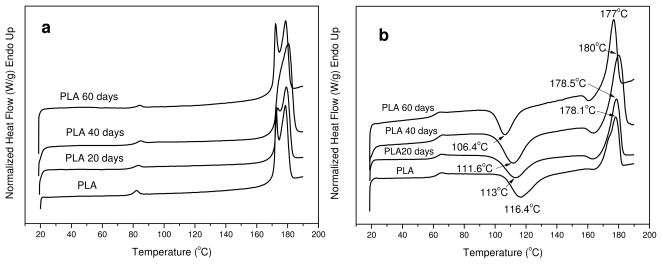
DSC scans of PLA ligament during hydrolysis. (**a**) First scan and (**b**) second scan after melt quenching.

All the samples after hydrolysis were also tested with DSC since the degree of crystallinity can change during this treatment. As can be seen, PLA ligament is a semicrystalline material with two melting peaks (Figure 10(a)). In the samples taken after hydrolysis at different times, two peaks are also visible but with some small differences. The melting points of these peaks at the first 40 days of hydrolysis have shifted to higher temperatures, but after 60 days they are shifted to lower values. However, most differences are in the values of heat of fusion. As can be seen in Table 3, the heat of fusion was gradually increased to higher values during hydrolysis. This was expected because the amorphous phase of aliphatic polyesters degraded more rapidly than the crystalline phase during hydrolysis. Thus, the degree of crystallinity (X_c_), as was calculated based on the enthalpy of fusion of 100% crystalline PLA, which is 93 J/g [45], increases during hydrolysis. However, after 60 days there is a stabilization, indicating that, after this time, the crystalline parts of PLA might also start to hydrolyze. This is because the molecular weight of the samples was drastically reduced at the same time.

The reduction in molecular weight during hydrolysis also has an effect on cold crystallization temperature (*T*_cc_) (Figure 10(b)). As can be seen, T_cc_ progressively shifts to lower temperatures by increasing the hydrolysis time. This is an indication that the samples crystallize faster than the initial sample; which was expected as it is well known that polymers with lower molecular weights have higher crystallization rates.

### 3.4. In Vivo Hydrolysis

**Figure 11:**
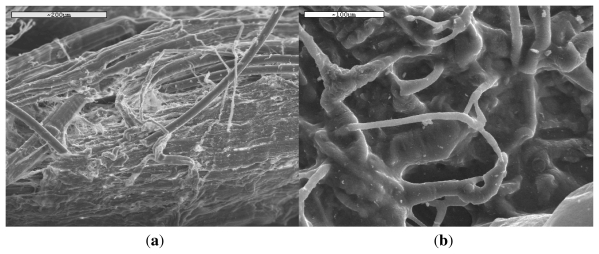
SEM micrographs of PLA ligament after 6 months of *in vivo* hydrolysis.

In addition to *in vitro* hydrolysis, the *in vivo* behavior of the PLA ligaments is also interesting. A time of 6 months after the addition of ligament in a human body was selected for tests, which is very short for a complete hydrolysis of PLA ligament. It was reported that PLA in the form of screws needs almost 60 months (5 years) during *in vivo* tests for complete disappearance of the screw [4]. In our case, a small sample was removed after 6 months from a human body and studied with SEM. The samples have been tasted without any cleaning. As can be seen from Figure 11, there are a lot of differences in the PLA ligament behavior. At some positions the ligament is almost intact (Figure 11(a)) and the fibers are well recognized due to their shape and mean diameter about 15 μm, exactly the same as the neat fibers (Figure 1), and in some other points the fibers have been covered with flesh (Figure 11(b)). This is a proof of high biocompatibility between the PLA ligament and the human body.

**Figure 12:**
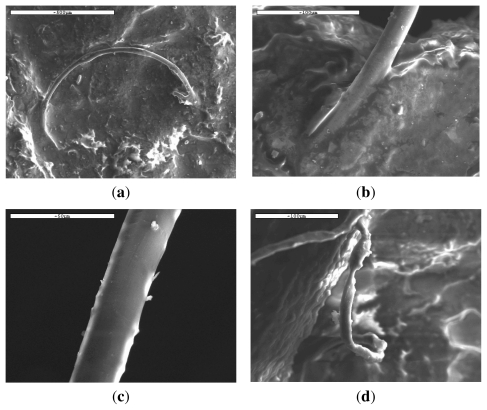
SEM micrographs of PLA ligament fibers after 6 months of *in vivo* hydrolysis.

Examining more carefully these fibers it can be seen that there are a lot of differences in their surfaces (Figure 12(a–c)) as well as in their shapes (Figure 12(d)). The surfaces are not as smooth as in the initial fibers indicating their alteration during *in vivo* hydrolysis. According to Pitt *et al.*, the *in vivo* degradation of similar polyesters like poly(dl-lactide) and poly(ɛ-caprolactone), proceeded in two stages: first there was only a decrease in molecular weight; subsequently the polymer experienced weight loss with an increase in the rate of chain scission [46]. Furthermore, from *in vivo* studies of PLA samples in the form of disks added in adult New Zealand white rabbits, small weight loss was found after 1-month implantation, corresponding to approximately 5% of its original weight and almost 30% after 6 months [27]. The number average molecular weight M_n_ of PLA was also reduced from 47,200 to 43,300 after 1 month and 3,600 g/mol after 6 months. In our case, it seems that the *in vivo* hydrolysis is slower than that reported previously, since only small differences are observed in the fibers’ appearance (Figure 12(a–c)). This may be due to the high molecular weight of the PLA used and short time of hydrolysis. If the rate was higher and molecular weight was dramatically reduced, some holes could be created on fibers surface due to elimination of soluble oligomers. This seems to be in agreement with the *in vitro* hydrolysis. However, from Figure 12(d) it is clear that some fibers are not cylindrical anymore and it seems that they have collapsed. From a previous work concerning the *in vivo* degradation of poly(a-hydroxy acids) derived from LA and/or GA monomers, it was found that all the intrinsically amorphous members of the family degrade hydrolytically and that degradation is faster in the centre than on the surface, at least for large devices [26]. Taking this study into account it can be concluded that the *in vivo* hydrolysis of the PLA ligament is also higher in the inner part of the fibers and lower on their surface. This may be a result of the higher crystallization rates of the PLA fibers’ surface during *in vivo* hydrolysis, as previously reported [4].

Further evidence for the small *in vivo* hydrolysis rate was provided from the molecular weight measurement that was performed on the removed sample. The Mn was reduced from approximately 120,000 g/mol to about 100,000–110,000 g/mol. Taking into account that the *in vivo* hydrolysis could be different between the fibers’ surface and their inner part, the molecular weight should be checked separately from these different areas. However, due to the small surface of the fibers, this was not possible and the molecular weight was measured from the whole sample. The small reduction in Mn after 12 months of *in vivo* hydrolysis can be attributed to the fact that the hydrolysis rate of PLA is taking place from the end of macromolecular chains and thus only small fragments could be removed. Thus, the molecular weight was almost unaffected. However, this is not in agreement with previous studies on *in vivo* hydrolysis in New Zealand white rabbits, where the rate was higher [27], or with the results from the *in vitro* hydrolysis of this particular sample discussed previously. This disagreement could be attributed to the higher temperatures during *in vitro* hydrolysis (50 °C). Also, as can be seen from the *in vitro* hydrolysis, the molecular weight reduction is very small for the initial 10–20 days, indicating that there may be an induction period after which the molecular weight decreases faster. Thus, it may be possible that 12 months is not time long enough for *in vivo* hydrolysis to cause a drastic reduction in the molecular weight of the sample.

**Figure 13:**
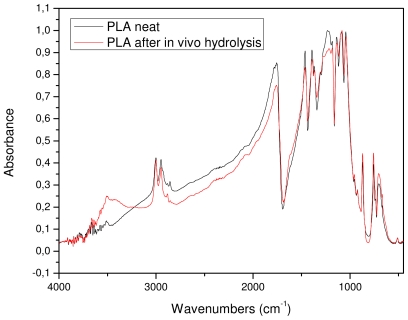
FTIR spectra of initial PLA ligament and after *in vivo* hydrolysis for 12 months.

The removed sample was also examined with FTIR spectroscopy in order to find any detectable differences in its chemical structure. As can be seen from Figure 13, the FTIR spectra of the initial sample and after *in vivo* hydrolysis are almost identical. This was expected since only small differences in molecular weight were recorded. The only detectable difference is the higher absorbance of the *in vivo* sample in the area of hydroxyl groups. However, this could not be attributed to the higher amount of hydroxyl groups that this sample contains but to the humidity that was absorbed by the sample.

**Figure 14:**
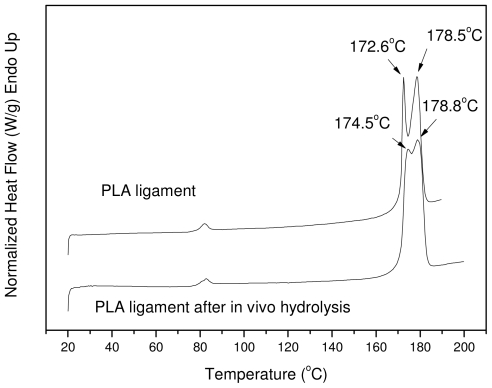
DSC thermograms of initial PLA ligament and after *in vivo* hydrolysis for 12 months.

The *in vivo* sample was also studied with DSC to see differences in its physical state and mainly in the degree of crystallinity; as found from the *in vitro* hydrolysis, the latter is one of the most notable changes. The DSC thermograms of the PLA Resorbaid sample initially and also after 12 months of hydrolysis are presented in Figure 14. As can be seen, the initial sample has two distinct peaks with maximum temperatures at 172.6 °C and at 178.5 °C. These two peaks are also recorded in the *in vivo* sample but at slightly different temperatures: 174.5 and 178.8 °C, respectively. Furthermore, in addition to the first peak shifting to higher temperatures, these two peaks tend to merge together into one peak. A similar behavior was also found in the sample after 40 days of *in vitro* hydrolysis (Figure 10(a)), where the sample showed a peak at higher temperatures than the initial sample. The heat of fusion in the case of the *in vivo* sample was also changed from 52.5 J/g to 57.8 J/g, which provides clear evidence that the degree of crystallinity increased from 56.45% to 62.15%, which is in accordance with the already detected changes during *in vitro* hydrolysis (Table 3). All these differences may be due to the *in vivo* hydrolysis of the amorphous part of the used reinforcement ligament or due to the annealing procedure of the polyester taking place during its insertion into the human body. The increased degree of crystallinity in comparison with the molecular weight of the initial sample may be the most probable reason for the low *in vivo* hydrolysis rate of the reinforcement ligament.

## 4. Conclusions

The PLA reinforcement ligament for applications in orthopaedics showed that it is a brittle material, since it breaks just after yielding, but that it has high mechanical strength, which is comparable to Fascia Latta. This is due to the high molecular weight of PLA used in the preparation of the ligament.

The material is semicrystalline and crystallizes during heating after melt quenching. Cold crystallization depends on the heating rate and shifts to higher temperatures as the rate increases. In general the crystallization rates are slow; however they were found to be faster than the crystallization rates measured in ordinary PLA samples. Cold-crystallization was analyzed using semi-empirical models for crystallization kintics. Tobin’s and Mo’s analyses were found to fit the cold-crystallization data quite well.

As was found from mass loss measurements, *in vitro* hydrolysis takes places slowly at the beginning of the phenomenon, but it is progressively accelerated after 30–40 days. However, after 80–100 days the rate seems to become progressively slower and thus the whole reduction seems to have an S-type behavior. Taking into account that the mass loss is very small at the initial stages, this is an indication that the macromolecular chains are first randomly broken down during hydrolysis and, when this happens excessively, soluble oligomers are formed resulting in a reduction to mass loss. The degree of crystallinity was also increased during hydrolysis, since the amorphous parts of PLA are more susceptible to hydrolysis.

The *in vivo* hydrolysis in the human body showed that the PLA reinforcement ligament is fully biocompatible and that, 6 months after implantation, most of its surface is covered with flesh. However, the hydrolysis rate is very small since the surface of the PLA fibers was almost unaffected, as found with SEM. This also was confirmed by molecular weight measurements that showed a small reduction, and from FTIR spectra indicating that the chemical structure of the reinforcement ligament remains unchanged after 12 months *in vivo* hydrolysis. The degree of crystallinity also increased during this time.

The high degree of crystallinity in combination with the molecular weight of the reinforcement ligament may be the most probable reasons for its low *in vivo* hydrolysis rate.

## Figures and Tables

**Table 1:** Results of Mo’s analysis for non-isothermal crystallization of Resorbaid material.

X (%)	*b*	ln *F*(*T*)
20	1.508	2.954
40	1.491	3.164
60	1.488	3.333
80	1.473	3.516

**Table 2:** Results obtained from the Tobin analysis for non-isothermal crystallization of Resorbaid material.

Heating Rate (°C/min)	*K_T_*	n_T_
20	0.30409	6.65
15	0.0881	7.04
7.5	0.0177	6.82
10	0.00356	6.92
7.5	5.19996 × 10^−4^	6.99
2.5	4.21697 × 10^−5^	6.4

**Table 3:** Melting points (T_m1_ and T_m2_), glass transition temperature (*T*_g_), cold crystallization temperature (*T*_cc_), heat of fusion (Δ*H*_m_) and degree of crystallinity (X_c_) of PLA during hydrolysis study.

Sample	*T*_m1_ (°C)	*T*_m2_ (°C)	*T*_g_* (°C)	*T*_cc_* (°C)	Δ*H*_m_(J/g)	X_c_(%)
PLA	172.5	178.5	61.5	116.4	52.5	56.45
PLA 20 days	173.4	179.2	62.2	113.0	55.3	59.46
PLA 40 days	174.3	180.5	62.1	111.6	59.6	64.08
PLA 60 days	172.2	178.8	61.1	106.4	61.2	65.80
PLA 120 days	172.0	178.2	61.0	102.5	60.7	65.26

* These data were taken from the second scan after melt quenching of the samples.
